# Age- and sex-related changes in children with and without generalized joint hypermobility: a two-year follow-up study

**DOI:** 10.1186/s12891-025-08684-y

**Published:** 2025-07-18

**Authors:** Oluwakemi Adebukola Ituen, Jacques Duysens, Gillian Ferguson, Bouwien Smits-Engelsman

**Affiliations:** 1 Department of Health & Rehabilitation Sciences, University of Cape Town, Cape Town, South Africa; 2 University of Uyo Teaching Hospital, Uyo, Akwa Ibom state Nigeria; 3 Motor Control Laboratory, Movement Control and Neuroplasticity Research Group, KU Leuven, Leuven, Belgium; 4 Physical Activity, Sport and Recreation, (PhASRec, focus area, Faculty Health Sciences, North-West University, Potchefstroom, South Africa

**Keywords:** Hypermobility, Children, Proprioception, Strength, Beighton

## Abstract

**Background:**

Joint hypermobility provides flexibility and is known to enhance motor performance but can also give rise to musculoskeletal complaints. There is evidence that young people are more flexible than older individuals, and females are more flexible than males. However, information about age- and sex-related changes in the range of motion (ROM) over time is scarce.

**Method:**

This study followed 126 children over two years; their ROM was measured three times with one year between measurements. The Beighton scoring system and goniometry were used to classify the children into normal mobile, mobile and hypermobile groups. The study included 56 males and 70 females. Mean age was 7.58 (6-9years), 8.58 (7-10years), and 9.60 (8-11years) years at time points 1, 2, and 3, respectively.

**Results:**

Joint hypermobility based on a Beighton score of 7–9 decreased from 25 to 13% to 6% in the last year. This was caused by a reduction of elbow and knee movement range but not by changes in finger joints or hamstring length. The pattern of decrease was very similar for boys and girls. Four children with hypermobility showed a large increase in ROM (> 10 degrees), of which 2 showed an increase in ROM at the elbow. Of the 53 children classified with normal mobility at measurement one 15 (28.3%) had one hypermobile joint (localized joint hypermobility).

**Conclusion:**

Children between 6 and 11 years of age get less flexible over 2 years. Mobility changes with age are comparable in boys and girls. Children who are not classified as generalized hypermobile can still have localized hypermobility. An increase in joint mobility is exceptional and may be a warning sign.

## Introduction

Children typically exhibit greater joint mobility than adults [1].When joints exceed the normal Range of Motion (ROM) due to ligamentous laxity, the condition is termed joint hypermobility [2]. Joint hypermobility can be: localized to a single joint, specific to a peripheral joint or occur throughout the body [3]. The latter, known as Generalized Joint Hypermobility (GJH), is the focus of this study. GJH is characterized by hypermobility in multiple joints, and is commonly observed in children [4], particularly among girls and individuals of African descent [5], as well as in athletes participating in sports that demand high flexibility [6]. GJH is typically asymptomatic, but when it becomes associated with musculoskeletal symptoms it is called Hypermobility Spectrum Disorder (HSD) [7]. The term HSD was suggested as a replacement for Joint Hypermobility Syndrome (JHS) by the International Consortium on Ehlers-Danlos Syndrome in 2017 for symptomatic, non-syndromic joint hypermobility [8]. While it is well established that adults are generally less mobile than children, preliminary data suggest that the loss of joint hypermobility may occur earlier in males than in females [9], potentially due to hormonal differences and variations in muscle mass development [10, 11]. However, data on sex-related changes in joint mobility in school-aged children are scarce.

In the past, GJH has been regarded as a benign trait associated with increased flexibility [3]. However, recent research has linked it to an increased risk of musculoskeletal symptoms such as pain, instability, impaired proprioception, and lower active joint stabilization [12, 13]. Despite these associations, the underlying mechanisms remain unclear, and there are currently no reliable biomarkers to diagnose symptomatic joint hypermobility [13].

While there is conflicting evidence about proprioceptive deficits in children with GJH, some studies do suggest impairments in symptomatic joint hypermobility [14, 15, 16]. These proprioceptive deficits could contribute to an increased risk of ligament or joint damage.

Muscle strength, particularly dynamic muscle strength, is another important area of concern [17]. While isometric strength deficits have been observed in adults with GJH [18], studies in children have yielded mixed results, with some showing no significant differences in strength compared to typically developing peers [15, 19]. The question remains if muscle strength in children with GJH is related to changes in joint hypermobility?

While deficits in proprioception and muscle strength have been reported in individuals with HSD, these findings do not necessarily indicate a causal relationship [20]. The question remains whether joints with impaired proprioception and muscle strength are more prone to musculoskeletal symptoms (such as pain or sprains), or if such symptoms lead to damage of mechanoreceptors and reduced muscle strength—potentially as a consequence of decreased physical activity [21]. If proprioceptive deficits and reduced strength are causal factors, they should precede the onset of musculoskeletal symptoms. A longitudinal design is therefore crucial to study these potential causal relationships.

The Beighton scale is the most widely used tool for assessing joint laxity, frequently employed in studies to evaluate the presence and severity of joint hypermobility [1]. It is scored dichotomously, with “1” indicating a positive sign of joint hypermobility and “0” indicating a negative sign [22]. The scale includes nine items and a score ranging from 0 to 9, reflecting the degree of generalized joint hypermobility, with higher scores indicating greater laxity. To improve clinical relevance and measurement accuracy, a revised protocol was introduced in 2011, incorporating goniometry to measure joint angles in the fingers, knees, and elbows [23].

Over time, some children diagnosed with GJH lose their joint hypermobility, while others remain hypermobile without developing symptoms [3]. Although numerous cross-sectional studies report a general decrease in joint hypermobility with age, few studies have provided joint-specific information on the trajectory of mobility changes and their potential implications for musculoskeletal health over time [21, 24, 25]. Such data are crucial for understanding the mechanisms behind the observed changes in joint laxity and the risk of damage to mechanoreceptors often seen in recurrent sprains [26]. This study also intends fill an important gap in understanding the relationship between proprioception, strength, and joint mobility over time, as previous studies have been cross-sectional.

This study aims to answer the following research questions:

1. Is there a significant change in joint mobility classification between the three measurements (one year apart) in children (ages 6–9 years to 8–11 years) as measured by the Beighton score?
2. Is there a significant change in the ROM in the 5th metacarpophalangeal (MCP-V) joints, elbows, and knees throughout the study?
3. Is there a significant change in the frequency of positive scores on item 5 (thumb to forearm) and item 9 (hands on the floor) over the course of the study?
4. Is the pattern of change in the total Beighton score and joint ROM different between boys and girls?
5. Are baseline measures of proprioception and muscle strength related to changes in joint mobility between measurement one and measurement three?

## Methods

### Study design

A longitudinal design with repeated measures was used to assess changes in joint hypermobility over time. Children were measured three times at one-year intervals. Ethical approval was obtained from the Human Research Ethics Committees of the University of Cape Town (HREC REF: 306/2021) and the University of Uyo Teaching Hospital (REF: UUTH/AD/S/96/VOL/XXI/524). The study was conducted in accordance with the Declaration of Helsinki [27].

### Participant description

Participants were recruited from schools in the Southern region of Nigeria. The convenience sampling method was used to select nine primary schools in Uyo. Next, at the level of the grades (classes), systematic sampling of the grades was done, followed by the recruitment of all the pupils aged 7–10 years in the selected classes as participants for the study. The study included children aged 6–9 years at the start of the study; this was to minimize attrition and ensure that the oldest participants would be 11 years old and still in primary school by the end of the 3-year study period. Written informed consent was obtained from parents, and assent was obtained from the children prior to inclusion. The following exclusion criteria were applied: (i) Children who have high-risk levels and poor safety as it pertains to physical activity; this was assessed using The Physical Activity Readiness Questionnaire (PAR-Q) for children [28]. (ii) Children who were limited in their ability to understand the testing instructions or the performance of the activities (e.g., cognitive impairment, gross motor impairment etc.) as reported by parents.

The sample size was determined using a power analysis for a repeated-measures design. A total sample size of 107 participants was estimated to achieve 90% power for detecting a medium effect size (d = 0.35) at an alpha level of 0.05. The G Power software version 3.1 was used for the calculation [29].

### Measurements

#### Anthropometric measures

Height (cm) and weight (kg) were measured using a measuring tape and weighing scale, respectively, with participants standing barefoot. Height was measured to the nearest 1 cm and weight to the nearest 100 g. Body Mass Index (BMI) was calculated and categorized using age- and sex-specific BMI percentiles as recommended by the World Health Organization [30].

#### Joint hypermobility

Joint hypermobility was assessed using the Beighton scoring system [31]. The Beighton scale includes nine items: four passive ROM assessments (performed bilaterally) and one active forward flexion task (Table 1). A score of 1 was assigned for a positive test, yielding a maximum total score of 9. The Beighton scale is considered a valid measure of joint hypermobility in children [23].

Participants were classified based on their Beighton scores into three mobility categories:

Normal-mobile (0–4 points), Mobile (5–6 points) and Hypermobile (7–9 points). These categories were used for the analysis, in line with a previous study by our group [24].

**Table 1:** Description of the Beighton scale items

Yes/No	Test	Bilateral test	Maximum score
Item 1	Passive dorsiflexion of the fifth metacarpophalangeal joint to ≥ 90 degrees	Yes	2
Item 2	Passive hyperextension of the elbow ≥ 10 degrees	Yes	2
Item 3	Passive hyperextension of the knee ≥ 10 degrees	Yes	2
Item 4	Passive apposition of the thumb to the flexor side of the forearm, while shoulder is flexed 90 degrees, elbow is extended, and hand is pronated	Yes	2
Item 5	Forward flexion of the trunk, with the knees straight, so that the hand palms rest easily on the floor	No	1
	Total score		9

#### Joint ROM

We applied the protocol by Smits-Engelsman et al. 2011 to a cohort of children, measuring joint ROM three times with one-year intervals to track changes over time [23]. This refined approach enhances the precision and sensitivity of the Beighton scale, allowing for more reliable assessments of joint hypermobility changes over time [24].

Joint range of motion was assessed in the MCP-V joint, elbow, and knee using a standardized joint hypermobility protocol [23]. A 360-degree goniometer (Lafayette Instrument Company, Lafayette, IN, USA) was used for the knee and elbow, while a small-arm goniometer was used for the MCP-V joint [24]. All measurements were taken bilaterally, with joint extension in the MCP-V joint, elbow, and knee recorded to the nearest 1 degree.

ROM values exceeding the Beighton criteria (e.g., elbow and knee ≥ 10° extension, MCP-V ≥ 90°) were considered indicative of “excess range of motion”.

#### Functional strength measure

**Fig. 1:**
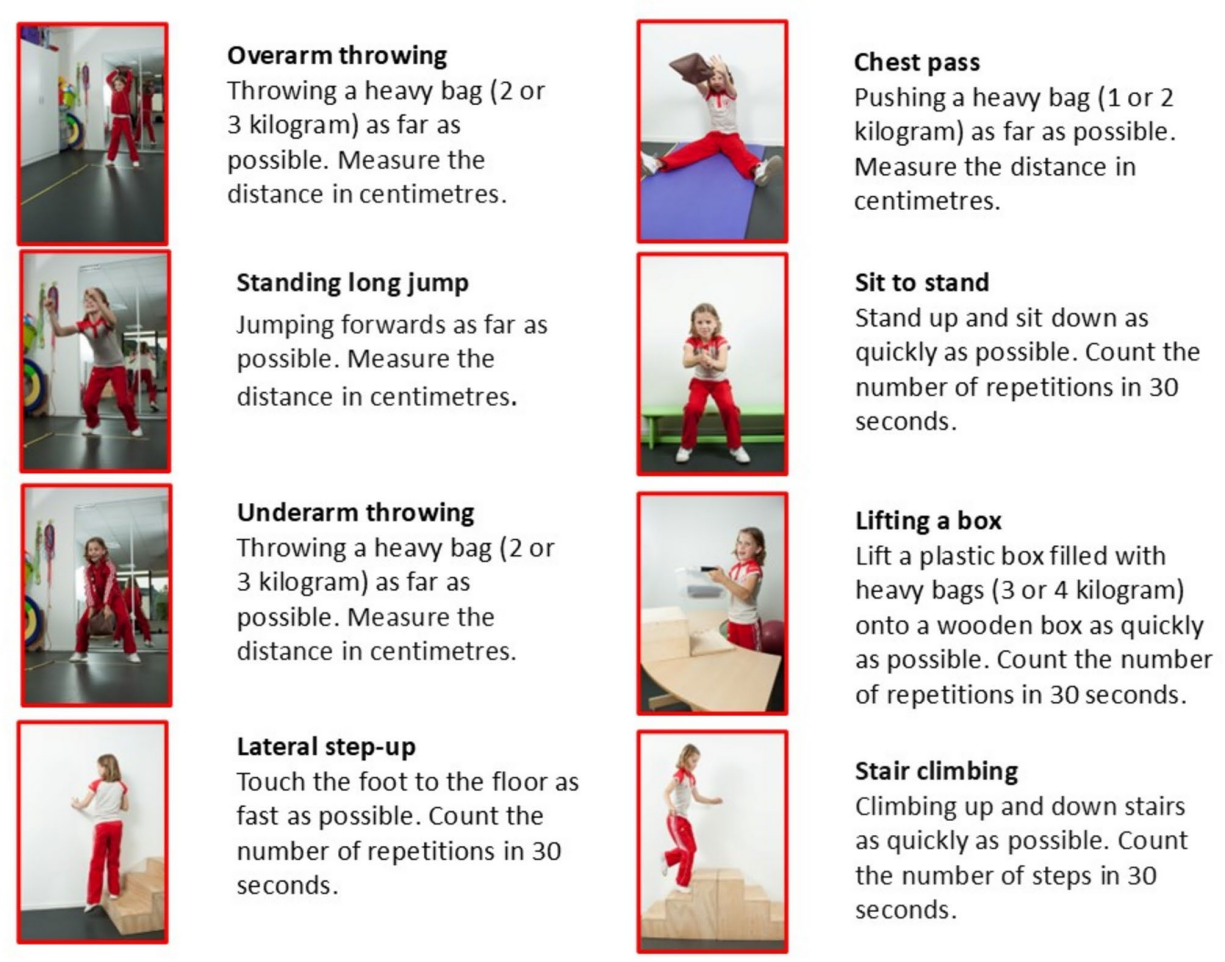
FSM items with short description [33]

The Functional Strength Measure (FSM) is a comprehensive, norm-referenced test for assessing functional strength in children [32]. The FSM measures both explosive power (single movement) and muscle endurance (number of repetitions in 30 s) [33]. The test includes eight items: four items tested the upper limbs (lifting a box with weights, overarm throwing, underarm throwing, chest press) and four items tested muscles of the lower limbs (standing long jump, sit-to-stand, lateral step-up, running up and down stairs) brief descriptions of the items are presented in Fig. 1 [33]. The standard protocol is a warm-up before the tests, followed by a practice trial and three rated trials. The best score from three attempts for each task was used in the analysis. The FSM has demonstrated high test-retest reliability (ICC = 0.91–0.94) [33]. This study used the FSM score at measurement 1 as a predictor in regression analyses.

#### Isometric strength

Maximum isometric muscle strength of the knee extensors, ankle extensors and flexors, and grip strength was assessed using a Hand-Held Dynamometer (HHD) (Lafayette Manual Muscle Testing System, Lafayette Instrument NY) [34]. The HHD has been shown to provide reliable strength measurements in children (ICC = 0.73–0.99) [33]. In our study, we followed the standardized protocol for testing as outlined by Beenakker et al. 2001. Knee extensor strength was measured with participants seated with knees at a 90° angle, feet unsupported. The HHD was placed on the anterior surface of the tibial shaft [35]. To measure ankle extensors and flexors, participants were positioned supine with the HHD placed on the sole of the foot for plantar flexion and on the dorsum for dorsiflexion [35]. For grip strength, the participant was seated, with the arm supported and the wrist in a neutral position. Participants were instructed to squeeze the HHD for three seconds [36]. The break method was used for all measurements, and the best of three trials was recorded and used for analysis. The isometric strength values at time one for knee, ankle, and hand muscles were used as predictors in regression analyses.

#### Proprioception

Proprioception was assessed using the Wedges test, following the standardized protocol outlined by Ituen et al. 2020 [15]. In addition to the Wedge heights used by Ituen et al. (3°, 6°, 9°, and 12°) we included Wedges of heights 1.5° and 4.5° similar to the study by Ebuka et al., 2023 [37]. The participants were asked to stand behind a table and instructed not to look at their feet, two Wedges of different or the same heights were placed under their feet randomly. The participants were asked to raise the arm on the side of the higher Wedge (right or left), or both arms if the Wedges height was equal. They were given 5 s to respond. A penalty was assigned for incorrect responses, with higher penalty scores indicating poorer proprioception. The total penalty score, which reflects proprioceptive accuracy, was used as a predictor in regression analyses.

### Data analysis

Data were analyzed using SPSS version 29. Descriptive statistics, including frequencies, percentages, means, and standard deviations, were calculated for participant characteristics (age, sex, BMI) and study outcomes (Beighton scale, ROM). Data were checked for outliers; for one child outliers were found in measurement 2 and this was taken out, data for one child was missing at measurement 2.

To assess changes over time in joint mobility (Beighton scale, ROM), repeated measures ANOVA was performed, with time (three measurement points) as the within-subject factor and sex (boys/girls) as the between-subject factor.

Cross-tabulation with Chi^2^ test was used to compare changes in the frequency of the scores for the dichotomous items on the Beighton scale.

To examine the relationship between changes in ROM and potential influencing factors, a stepwise multiple linear regression was used to predict changes in joint mobility over time using baseline measures of strength (functional and isometric) and proprioception as predictors. The data of the predictors that were entered in the regression analysis showed normal distribution. A significance level of α = 0.05 was set for all statistical tests, and 95% confidence intervals were used.

## Results

### Participants’ data

A total of 126 children participated in the study, including 56 males (44.4%) and 70 females (55.6%). None of the children were excluded using the mentioned criteria. At the start of the study, 94 (38.4%) children had a total Beighton score of 0–4 (normal mobile), 24 (9.8%) children scored 5–6 (mobile) and 8 (3.3%) children scored 7–9 (hypermobile). The mean age and BMI at measurements 1, 2, and 3 are presented in Table 2. Growth was observed over the study period: children became heavier (F (2,122) = 144.85, *p* < 0.001) and taller (F (2,122) = 561.55, *p* < 0.001), with post-hoc analyses showing significant increases between all measurement points. The rate of growth, however, was higher for girls than for boys (Interaction: Time × Sex (F (2,122) = 6.35, *p* = 0.002)).

**Table 2:** Participants’ demographic data per measurement

Demography	Measurement 1 Mean (SD)	Measurement 2 Mean (SD)	Measurement 3 Mean (SD)
Age (years)	7.58 (0.88)	8.58 (0.88)	9.60 (0.88)
BMI (kg/m^2^)	17.76(2.28)	15.04 (2.21)	15.07(2.47)
Weight (kg)	22.94(4.62)	25.68 (5.51)	27.37(6.02)
Height (cm)	124.4(7.37)	130.12 (7.28)	134 (8.61)

### Changes in mobility classification

**Fig. 2:**
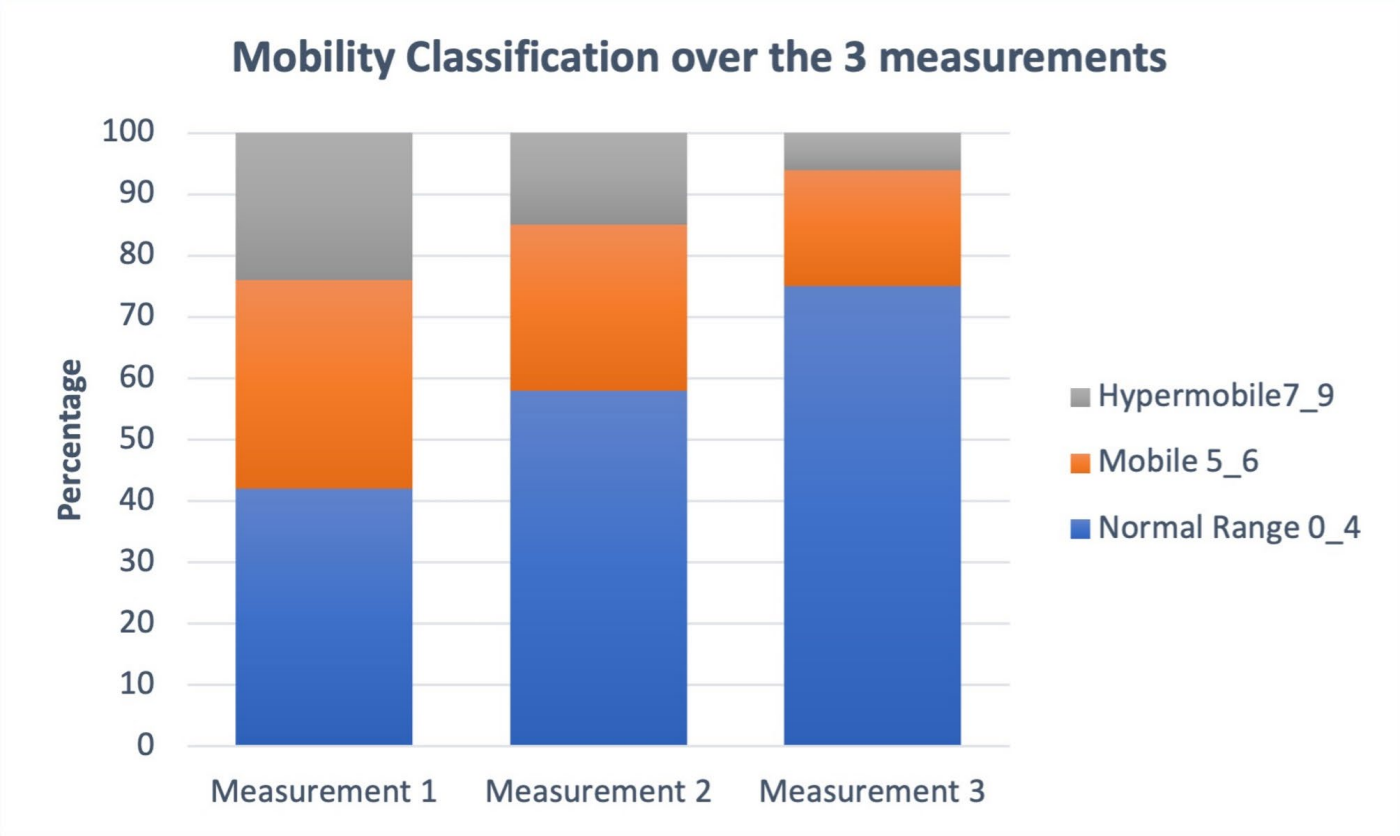
Changes in the frequency (percentage) per Mobility classification based on Total Beighton score over the three measurements

The frequency of changes in Beighton classification across the three measurements was assessed. The percentage of children classified as hypermobile decreased over time (Fig. 2). Large changes in mobility classification were observed, with a trend towards an increase in children classified within the Normal range and a decrease in those classified as Mobile and Hypermobile over the three measurements.

### Total beighton score

Means and SD of the total Beighton score per measurement are shown in Table 3.

**Table 3:** Mean (SD) of total Beighton score over three measurements per mobility classification

Mobility classification based on Beighton score	Total Beighton score 1st measurement	Total Beighton score 2nd measurement	Total Beighton score 3rd measurement
Normal mobile (0–4) Mean (SD)(*n* = 53)	3.15 (0.99)	2.82 (1.38)	2.64 (1.52)
Mobile (5–6) Mean (SD) (*n* = 42)	5.48 (0.51)	4.39 (1.77)	3.43 (1.47)
Hypermobile (7–9) Mean (SD) (*n* = 31)	7.48 (0.57)	5.83 (1.44)	5.03 (2.01)

n = Number of participants

### Changes in ROM and beighton item scores

**Fig. 3:**
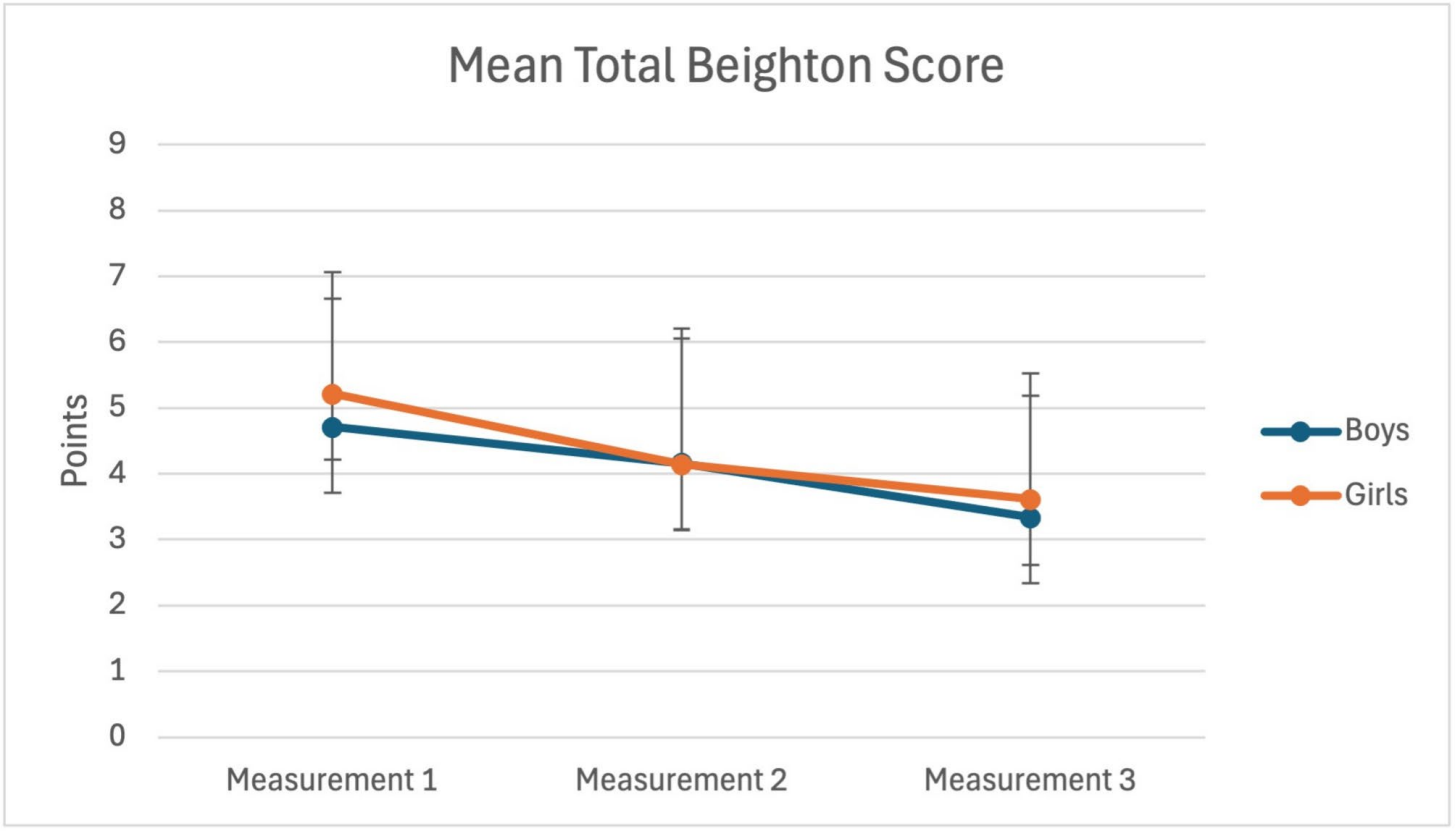
Changes in ROM over the three measurements per Mobility group. Item 1 showed no decrease in ROM, while hyperextension decreased in all groups in the elbow and knee. Data are shown of the Right joints. Degrees for MCP-V depict the measured hyperextension > 90° for elbow and knee > 10 degrees hyperextension

**Fig. 4:**
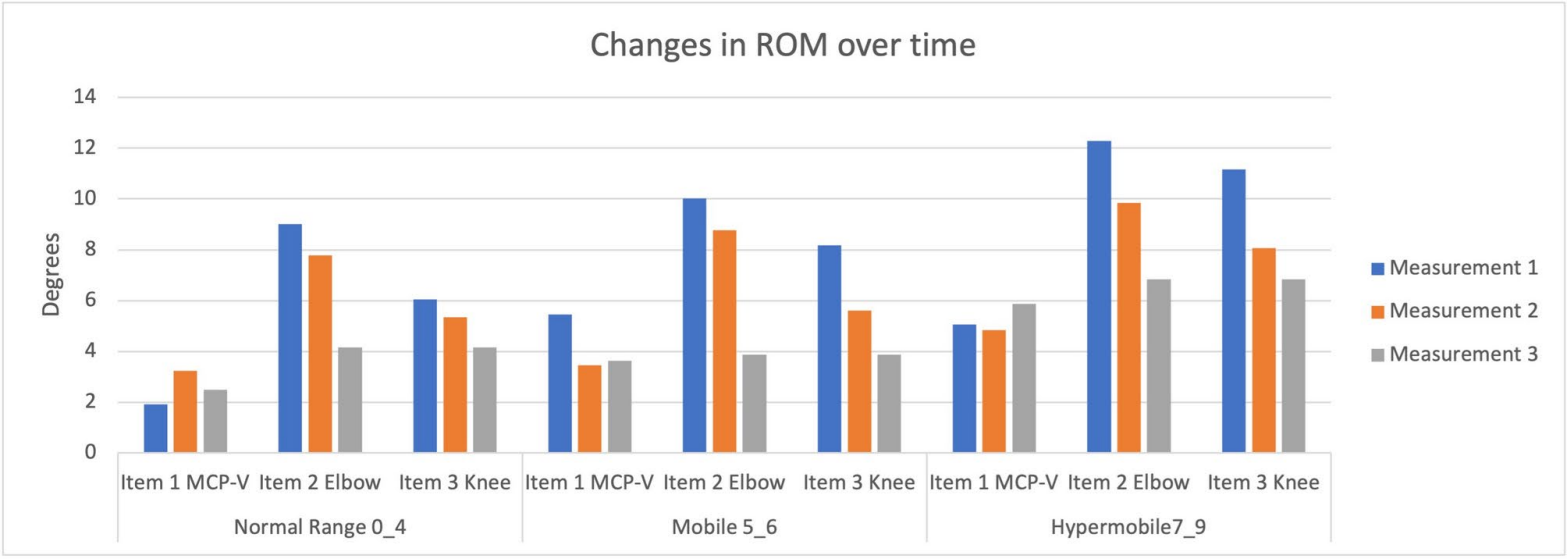
Total Beighton score over three measurements for boys and girls. Error bars depict 95% confidence intervals

Changes in ROM across the various joints were analyzed (Table 4). ROM for the MCP-V (Item 1) did not show significant changes over time, but knee and elbow ROM showed significant decreases (*p* < 0.001) (Fig. 3). Interestingly, only the left knee change in ROM was greater in girls compared to boys (7.3° vs. 6.1°). Significant interactions between time and sex were observed for right elbow ROM (F(2,121) = 5.46, *p* = 0.005), with girls showing a decrease in elbow ROM across each measurement. At the same time, boys exhibited no changes between time two and time three. Repeated measures analysis of the total Beighton score indicated a large decrease over time (F (2,121) = 36.90, *p* < 0.001), with significant post-hoc differences between each consecutive measurement (Table 4). No significant main effect of sex was found (*p* = 0.34), nor was there any interaction between time and sex (*p* = 0.16) (see Table 4; Fig. 4).

**Table 4:** Statistics for changes over time for the total Beighton score and per item: repeated measure ANOVA for ROM (items 1, 2 and 3) and changes in frequency of items 4 and 5 (Chi^2^)

Variable	Main effect Time	Main Effect Sex	Interaction Time x Sex	Post hoc between measurement 1 2and 3
Total Beighton	F(2,121) 36.90, *p* = 0.001	*p* = 0.34	*p* = 0.16	1–2: *p* = 0.001 2–3: *p* = 0.001 1–3: *p* = 0.001
Item 1 MCP-V R ROM	p 0.89	*p* = 0.19	*p* = 0.21	
Item 1 MCP-V L ROM	*p* = 0.71	*p* = 0.44	*p* = 0.86	
Item 2 Elbow R ROM	F(2,121) 28.20, *p* = 0.001	*p* = 0.11	F(2,121)5.46 *p* = 0.005 Girls decreased every year, boys no change between 2–3	1–2: *p* = 0.001 2–3: *p* = 0.001 1–3: *p* = 0.001
Item 2 Elbow L ROM	F(2,121) 23.51, *p* = 0.001	*p* = 0.50	*p* = 0.27	1–2: *p* = 0.001 2–3: *p* = 0.40 1–3: *p* = 0.001
Item 3 Knee R ROM	F(2,121) 44.07, *p* = 0.001	*p* = 0.22	*p* = 0.70	1–2: *p* = 0.001 2–3: *p* = 0.001 1–3: *p* = 0.001
Item 3 Knee L ROM	F(2,121) 34.69,*p* = 0.001	*p* = 0.023 Girls more mobile	*p* = 0.67	1–2: *p* = 0.001 2–3: *p* = 0.001 1–3: *p* = 0.001
Item 4 Thumb R	Measurement 1–2: z-2.78, *p* = 0.005 Measurement 2–3: z-5.01, *p* = 0.001 Measurement 1–3: z-5.43, *p* = 0.001	*p* = 0.61 *p* = 0.24 *p* = 1.00		
Item 4 Thumb L	Measurement 1–2: z-1.04, *p* = 0.29 Measurement 2–3: z-5.01, *p* = 0.001 Measurement 1–3: z-5.19, *p* = 0.001	*p* = 0.81 *p* = 0.16 *p* = 0.83		
Item 5 Hands on floor	Measurement 1–2: ns *p* = 0.47 Measurement 2–3: ns *p* = 0.27 Measurement 1–3: ns *p* = 0.06	*p* = 0.26 *p* = 0.84 *p* = 1.00	Measurement 1 88.9% score negative Measurement 2 84.9% score negative Measurement 3 81.7% score negative	

ROM = Range of Motion

### Changes in excess ROM between measurements 1 and 3

The change in excess ROM (sum over the six measured joints) between Time 1 and Time 3 was calculated. The mean change was 12.4°, (range + 31° to -78°). Notably, 73.1% of children became less mobile, while 19% became more mobile, and 7.9% did not show any change in ROM. (See Table 5).

**Table 5:** Frequency of changes in ROM (degrees) per mobility classification

Change ROM (degrees)	Normal Range 0_4	Mobile 5_6	Hypermobile 7_9	Total
Large increase (31 − 10)	4	2	4	10
Small increase (9 − 3)	8	4	2	14
No difference (-2 till + 2)	5	2	3	10
Small decrease (-9 to-3)	11	6	5	22
Large decrease (-10 to-29)	24	19	10	53
Very large decrease (30–78) Total number of Children	1 53	9 42	7 31	17 126

ROM = Range of Motion

ROM decreases over time in all mobility groups. There was a significant difference in the change in excess ROM between mobility groups (F(2,123) = 3.37, *p* = 0.038). Excess ROM was 10 degrees for children in the mobile group and 20 degrees for children in the hypermobile group, summed over the 6 joints in year 1. At the second Measurement point, the children classified as mobile in year 1 no longer had a mean excess ROM. The mean degrees range of motion in excess of the Beighton cut-off values in hypermobile children dropped from 20^°^ at Measurement 1 to 9^°^and 6^°^ more than the Beighton criteria at Measurement point 2 and 3, respectively (Table 6).

**Table 6:** ROM in excess of the Beighton cut-off values (MCP-V > 90, elbow and knee > 10 degrees hyperextension) for the three mobility groups over the three measurements. Negative scores indicate degrees less than the Beighton criteria

		Excess ROM	
Mobility Classification	Measurement 1 Mean (SD)	Measurement 2 Mean (SD)	Measurement 3 Mean (SD)
Normal score 0–4	-3.5 (9.9) ^°^	-5.3 (10.2) ^°^	-11.6 (11.6) ^°^
Mobile score 5–6	10.7 (9.9) ^°^	-1.0 (10.0) ^°^	-5.9 (10.1) ^°^
Hypermobile score 7–9	20.2 (13.2) ^°^	9.4 (11.3) ^°^	6.0 (14.2) ^°^

ROM = Range of Motion

On examination of individual joints, we noted that the hypermobile and mobile groups exhibited a greater decrease in knee ROM compared to children in the normal mobility group (Right Knee: F (2,123) = 6.73, *p* = 0.002; Left Knee: F (2,123) = 4.00, *p* = 0.021).

### Clinical subgroups of interest

Two clinical subgroups were of particular interest in this study: children with normal mobility who still exhibited local excess ROM, and children with hypermobility who showed an increase in ROM during the study period. Of the 53 children classified as normal mobility at Time 1, 15 (28.3%) had excess ROM, mainly in the MCP-V and elbows. Interestingly, none of these children could perform the hands-on-floor or thumb-to-forearm tests, both of which are Beighton criteria for hypermobility.

Among the 31 children classified as Hypermobile at Time 1, only 4 (12.9%) showed a large increase in ROM (> 10 degrees), during the study (Table 5) which was again caused by MCP-V. Only two children showed increased ROM at the elbow, none at the knee.

### Relationship between changes in mobility and proprioception/strength

To investigate the factors associated with changes in mobility, all strength items and total penalty score were entered as predictors in the regression analysis; this model explained 15% of the variance in changes of the excess ROM. Due to the lack of association, the model did not reach significance (*p* = 0.14); neither did the regression models where change in knee range of motion or change in total Beighton score were entered as dependent variables.

## Discussion

Although joint hypermobility has been widely studied in cross-sectional research, there is a notable gap in longitudinal studies exploring how joint mobility changes over time, particularly in children [6, 19]. The findings of our study revealed a significant reduction in joint mobility among children aged 6–9 years at the first measurement and 8–11 years at the last measurement. No differences in the changes in mobility classification were found between boys and girls. Knee and elbow ROM showed significant decreases over time, with a significant interaction between time and sex for the right elbow ROM. Neither the strength nor proprioception were significant predictors for changes in the total score of the Beighton.

Our longitudinal data confirm that joint mobility had reduced by the 3rd measurement (8–11 years). The systematic review by Sobhani-Eraghi et al. 2020 reported that a decrease in the prevalence of joint hypermobility was associated with an increase in age, some authors attributed this to tissue maturation [38, 39]. The large changes in joint mobility have provided some evidence that joint mobility decreases faster at a younger age as suggested in the literature [40]. We did not find a sex difference in joint mobility, some previous authors have tried to associate this conflicting trend to the peculiar characteristics of the ethnicity or geographical location under evaluation [39].

### The Beighton scale and joint mobility assessment

In our study, we supplemented the Beighton score with goniometric measurements of joint ROM, which allowed us to calculate the excess range of motion for each joint and showed it to be a more sensitive measure for change. While the general trend showed that most children became less mobile over time, we found that 19% became more mobile. This supports the proposed diagnostic framework by Tofts et al. 2023 that views diagnosis as fluid and children’s joint mobility status can change [9]. Interestingly, the increased ROM was predominantly seen in the little finger (Item 1), across all three mobility groups. Upon further examination of the hypermobile children, we found that none experienced increased knee joint ROM. However, a small number (2 and 4 children) showed increased ROM at the right and left elbows, respectively. These children did not have any deficiency in proprioception and muscle strength related to changes in joint mobility, suggesting that the increase in ROM might (still) be functionally benign [41].

An important consideration in this study is the specific behavior of individual Beighton items over time. Two of the five items did not follow the general trend suggesting a decline in hypermobility as children age. Notably, the ROM of the little fingers (Item 1) did not change significantly over time. Item 5, which assesses the ability to place the hands flat on the floor, has also been identified as an outlier. It was the only item showing increased positive scores (11.1%, 15.1%, to 18.3% for Measurement 1, 2, and 3, respectively). This finding is consistent with our earlier work, which highlighted the unique nature of Item 5 compared to other Beighton items [18, 39]. Unlike other Beighton items, which measure joint mobility, Item 5 reflects muscle length rather than joint laxity [42]. This distinction is important because it raised the question of whether the scores on this item would change in the same way as those for other Beighton items as children age, and our results showed that it did not.

While the pattern of change in the total Beighton score and joint ROM was very similar for boys and girls, we observed one notable sex difference: at the elbow, girls experienced a significant decrease in ROM at each measurement point, whereas boys showed no significant change between measurement 2 and 3. This suggests that, while the overall trend of reduced mobility is consistent across sexes, girls may experience more pronounced changes at specific joints over time.

### Implications of strength and proprioception for increased mobility

While increased joint mobility is not inherently problematic, it may contribute to chronic pain, impaired motor function, and lower levels of physical fitness [43]. One hypothesis is that the laxity of ligaments in hypermobile joints leads to abnormal biomechanics, compromising joint stability and increasing the risk of injury [44]. Altered biomechanics can result in improper loading of joints, which may cause micro-traumas that damage ligaments, capsule, and mechanoreceptors responsible for proprioception, leading to decreased joint stabilization, and motor control [45]. Although GJH has been associated with a higher risk of musculoskeletal injury, only a small proportion of individuals with GJH develop persistent musculoskeletal pain or meet the criteria for connective tissue disorders [46, 47]. Our findings support the idea that muscle strength and proprioception are not significant factors in explaining changes in joint mobility during childhood. In a cross-sectional study of GJH in children aged 2.5–12 years, even though their motor development was delayed, no association was found between GJH and motor development [48]. Also, in a study including children and adults with GJH by Jensen et al. 2013, an association was found between Beighton score and performance in terms of force steadiness and co-activation ratio in adults with GJH during knee flexion tasks, but this association was absent in children with GJH. This suggests that, in the early years, other factors—such as natural age-related changes in ligamentous laxity and motor development—might play a more prominent role in the changes in joint mobility. Although females exhibit increased joint laxity during puberty when compared to males, research has not been to establish a clear association between joint laxity and puberty [49]. Furthermore, irrespective of presence of joint laxity, females have significantly demonstrated significant muscular deficits following puberty when compared to their male counterparts [49, 50]. Interestingly, Seger, and Thorstensson 2000, found in their study of males and females post puberty that muscle strength can also be a function of muscle action type (eccentric or concentric), as females demonstrated the tendency to increase their eccentric strength more than their concentric strength [50]. Due to lack data on the pubertal status of our study participants we were unable to test the influence of puberty on our participants’ joint mobility, muscle strength and proprioception over time.

### Clinical significance

The clinical significance of our study lies in the inclusion of goniometry with the Beighton scoring system to identify children with GJH. Beyond the dichotomous identification of joint hypermobility by the Beighton scoring system, goniometry gives an indication of the severity of joint hypermobility, thus increasing the sensitivity of identifying children with GJH who may possibly develop musculoskeletal symptoms later in life, leading to early interventions and prevention of complications. In addition, the use of goniometry is also significant to identify Localized Joint Hypermobility, since the joint micro and macro trauma may lead to a series of musculoskeletal symptoms such as joint pain, dislocation, sprain and strain [51]. The onset of puberty induces changes in joint flexibility and strength but may also influence the incidence and severity of injuries especially in females [50, 52]. Thus, clinically assessing joint hypermobility and strength in maturing adolescents presenting with musculoskeletal symptoms is important.

### Strengths and limitations of the study

The longitudinal design of our study is a strength, as it allows for the examination of both age-related changes and individual variability in joint mobility. While the one-year time frame between measurements provides meaningful data on the progression of mobility over time, it also introduces potential limitations. For example, retesting effects—such as changes in motivation or a learning effect—could have influenced the scores. We acknowledge that the generalization of our study can be limited due to the selection of the sample. No data were collected on participants’ pubertal status. Thus, it could not be tested if the onset of puberty affected the decline in the ROM over time. A consideration for future study may be to examine if hormonal changes play a role in the onset of musculoskeletal symptoms in GJH.

## Conclusion

In conclusion, generalized joint hypermobility (GJH) is common in children, but the frequency of hypermobility decreases with age. Our study highlights the exceptional occurrence of increased ROM in the knees and elbows, which is not typical for children as they grow older. Additionally, our results suggest that muscle strength and proprioception do not significantly influence changes in joint mobility during childhood. However, these factors may become more relevant in adolescence and adulthood, particularly in the context of functional activities and injury prevention.

The effects of excess range of motion on musculoskeletal health are complex and require further investigation, particularly in adolescents and young adults. Early diagnosis and management of children with GJH are crucial to prevent long-term complications and to improve overall well-being, particularly as they transition into adulthood.

## Data Availability

The datasets used and/or analyzed during the current study are available from the corresponding author upon reasonable request.

## Abbreviations

ROM: Range of Motion
BMI: Body mass index
GJH: Generalized Joint Hypermobility
HSD: JH Joint Hypermobility
MCP: Metacarpophalangeal
FSM: Functional Strength Measurement
WHO: World Health Organization
HHD: Hand Held Dynamometer
HCTD: Heritable Connective Tissue Disorder
HSD: Hypermobility Spectrum Disorder
